# Klebsiella pneumoniae endogenous endophthalmitis presenting as orbital cellulitis

**DOI:** 10.3205/oc000119

**Published:** 2019-08-20

**Authors:** Benjamin Kambiz Ghiam, Paul Israelsen, Angeline Wang, Seanna Grob, Mohammad Riazi Esfahani

**Affiliations:** 1 Oakland University, William Beaumont School of Medicine, Rochester, United States; 2 Gavin Herbert Eye Institute, University of California, Irvine, United States

**Keywords:** endogenous endophthalmitis, Klebsiella pneumoniae, liver abscess, orbital cellulitis

## Abstract

This report describes the first documented case of *Klebsiella pneumoniae* endogenous endophthalmitis with concurrent orbital cellulitis in a patient without a liver abscess. A 34-year-old, caucasian male with a history of intravenous drug abuse and type 1 diabetes mellitus was transferred from an outside hospital for progressively worsening pain and swelling in the right eye. Careful history, physical examination, and imaging were consistent with a diagnosis of endogenous endophthalmitis with concurrent orbital cellulitis. Vitreous cultures were positive for *Klebsiella pneumoniae*. Despite aggressive and early intervention with antibiotics and vitrectomy, the patient’s condition continued to worsen and evisceration was required to adequately control the infection. *Klebsiella pneumoniae* endogenous endophthalmitis with concurrent orbital cellulitis is a rare and often blinding infection, despite appropriately aggressive intervention.

## Introduction

*Klebsiella pneumoniae* endogenous endophthalmitis (KPEE) is a devastating infection that is uncommon in western countries, while increasingly prevalent in Asian countries [1], [2]. KPEE classically occurs in patients with systemic disease and underlying immunosuppression, including pyogenic liver abscesses or diabetes mellitus [3], [4]. Despite treatment with intravenous and intravitreal antibiotics, visual outcomes are almost uniformly poor in KPEE, with most patients having a final visual acuity of counting fingers or worse [4], [5]. A particularly severe and rare manifestation of bacterial endophthalmitis is the concurrent presentation of orbital cellulitis [6]. The present study examines a case of *K. pneumoniae* endogenous endophthalmitis presenting initially as orbital cellulitis in an intravenous drug user.

## Case description

A 34-year-old, caucasian male was transferred from an outside hospital to a university hospital for progressively worsening pain in the right eye. The patient had a history of intravenous drug abuse, type 1 diabetes mellitus, multiple arm and leg abscesses requiring incision and drainage, and multiple hospitalizations for extremity cellulitis. Five days prior to being transferred to our facility, the patient noted discomfort of the right eye and was started on a topical antibiotic drop for a corneal abrasion. Over the next few days, the patient developed severe right eye pain with limitation of eye movements, right upper and lower eyelid swelling, and worsening of vision. He denied any history of ocular surgery, eye trauma, or recent nasal congestion.

**Figure 1:**
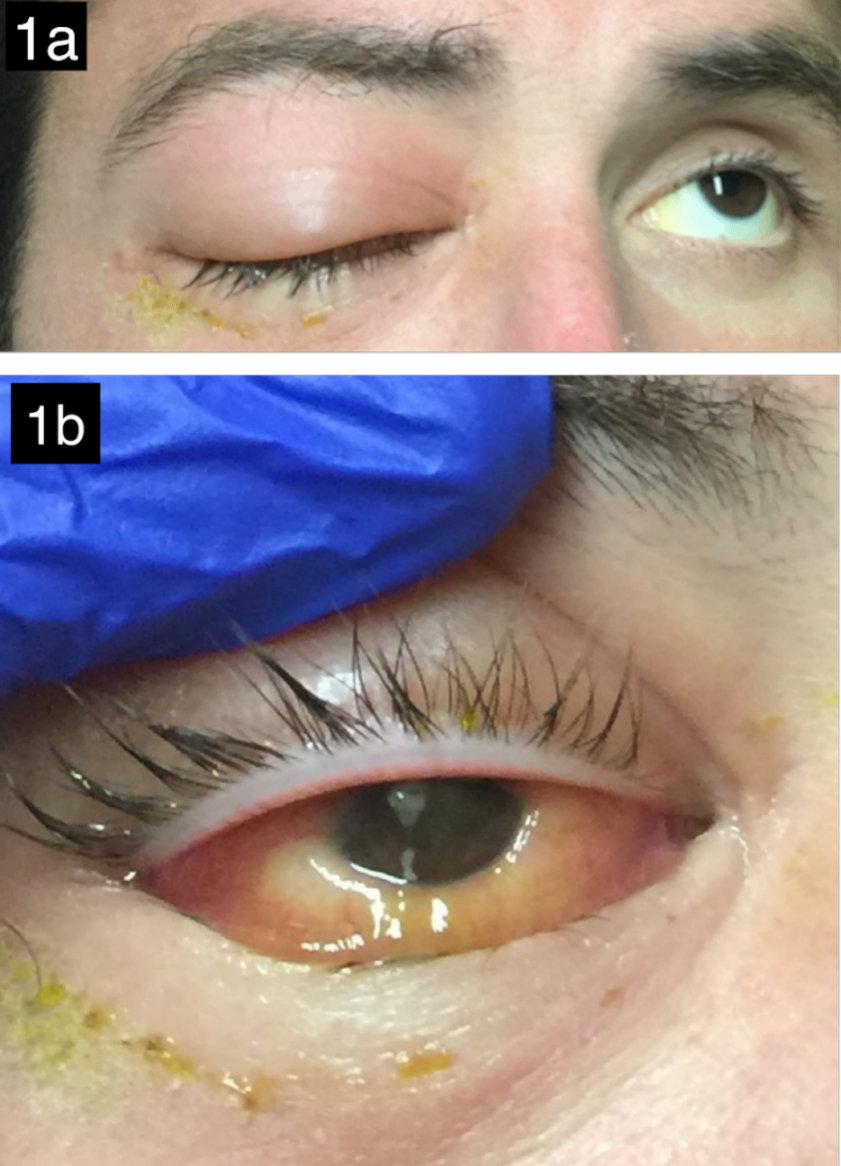
External photograph displaying moderate edema and erythema of the right upper and lower eyelids, as well as complete ptosis of the right upper eyelid (a). The left eye is unremarkable. External photograph of the patient’s right eye being manually opened (b). There is marked chemosis of the conjunctiva and mucous strands on the cornea. A pupillary hypopyon is partially visible.

On initial presentation, the patient was noted to be alert, afebrile, and normotensive. The visual acuity (VA) was light perception only in the right eye and 20/20 in the left eye. Intraocular pressure (IOP) was 31 in the right eye, and 14 in the left. In the right eye, complete ptosis was present and motility was severely limited in all directions. There was moderate proptosis of the right eye, tense edema and erythema of both the right upper and lower eyelids (Figure 1a (Fig. 1)). The patient also had significant chemosis (Figure 1b (Fig. 1)), fibrin over the pupil and a 0.5 mm hypopyon. There was no corneal abrasion or infiltrate. Eyelids were tense and it was difficult to open the eyelids. The vitreous and fundus were not visible at the slit lamp.

**Figure 2:**
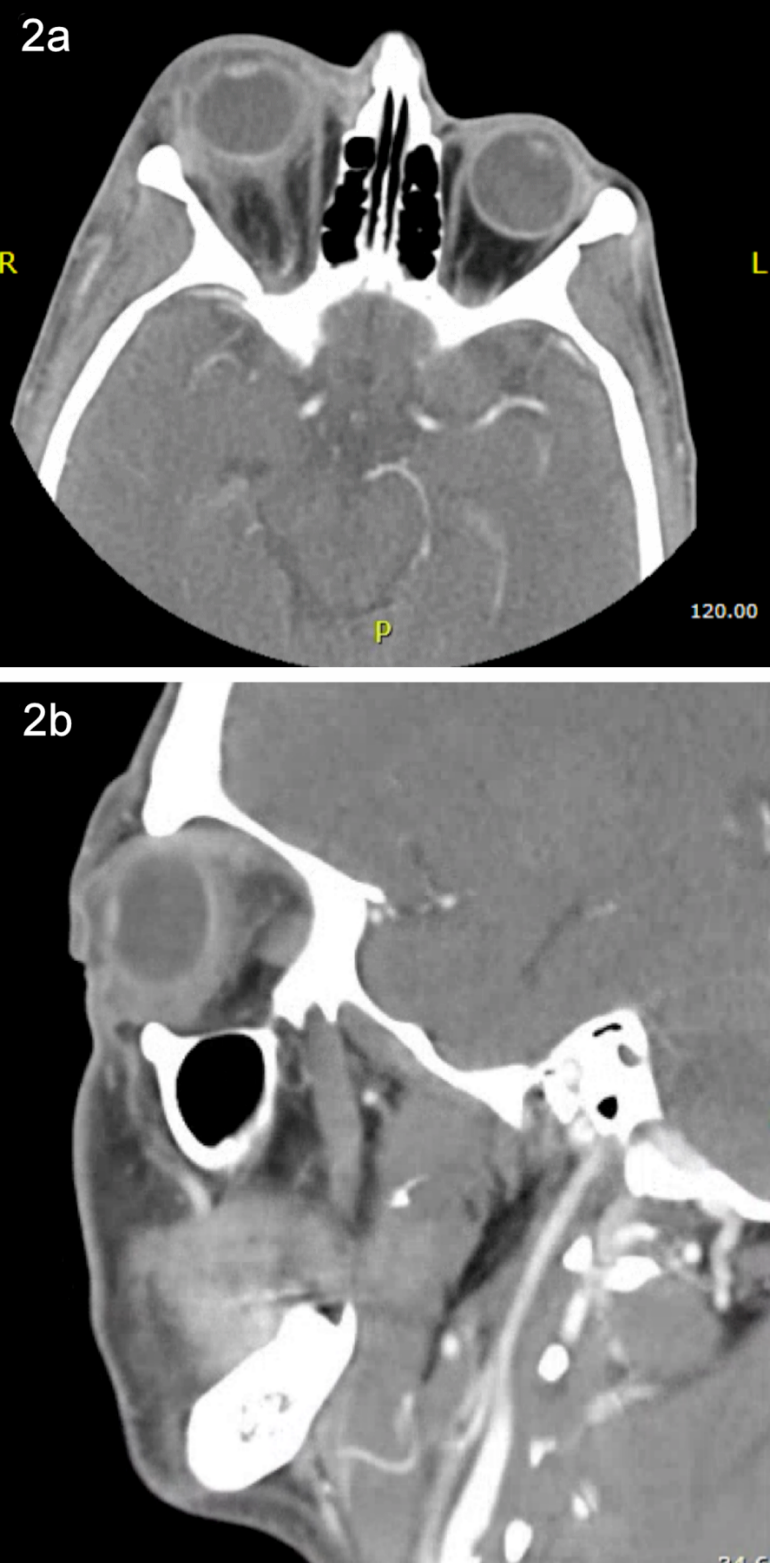
Axial (a) and sagittal (b) computed tomography (CT) post-contrast images showing proptosis of the right eye with orbital fat stranding focused primarily around the globe. There is no evidence of sinus disease or subperiosteal abscess.

**Figure 3:**
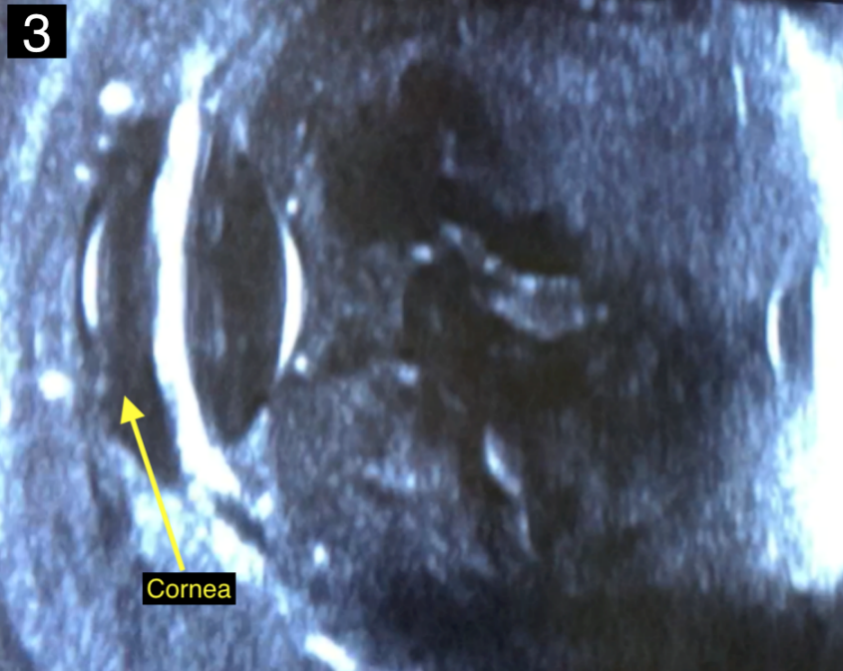
Ultrasound image of the right eye demonstrating dense hyperechoic cellular debris within the vitreous cavity suggestive of vitritis

The white blood cell count was 12,800 cells/mm^3^, blood glucose was 331 mg/dl, and liver transaminases within normal limits. A computed tomography (CT) scan of the orbits showed right-sided orbital cellulitis with significant fat stranding centered around the globe and no significant sinus disease (Figure 2 (Fig. 2)). Ocular ultrasound demonstrated hyperechoic material in the vitreous cavity consistent with vitritis (Figure 3 (Fig. 3)). Abdominal ultrasound revealed mild hepatomegaly without a hepatic abscess. 

Due to the severity of the exam findings and the need for intravenous antibiotics, the patient was admitted to the hospital. A vitreous tap and intravitreal injection of vancomycin and ceftazidime were performed immediately and the vitreous sample was sent for culture. Intravenous vancomycin and piperacillin/tazobactam were also started. Ocular antihypertensive medications were also administered topically at regular intervals. 

The following day, the right eye IOP was 50, and the patient’s proptosis and pain had worsened. Gram stain of the vitreous sample obtained the previous day showed numerous gram-negative rods. Vitreous tap and intravitreal injection of ceftazidime was performed. Due to the worsening exam findings and elevated IOP, lateral canthotomy and cantholysis were performed. Despite this procedure, the IOP remained persistently elevated at 45. 

Afterwards, the VA worsened to poor light perception and the patient underwent a right pars plana vitrectomy with lensectomy and silicone oil injection. Dense vitritis, widespread retinal necrosis, and nasal rhegmatogenous retinal detachment were noted intraoperatively. Vitreous cultures grew the bacteria *Klebsiella pneumoniae*. A presumptive diagnosis of *K. pneumoniae* endogenous endophthalmitis with orbital cellulitis was made. The infectious disease team was consulted, and the intravenous antibiotic regimen was narrowed to high-dose cefepime. On the ninth day of admission, due to persistent proptosis and pain in the setting of NLP vision, the patient underwent evisceration of the right eye. A large scleral perforation was identified intraoperatively with necrotic tissue surrounding it. Scleral biopsy cultures later grew *K. pneumoniae*. After evisceration, the patient improved rapidly and was eventually discharged on oral moxifloxacin.

## Discussion

Specific virulence factors have been implicated in the pathogenicity of *Klebsiella* sp. The organism is an enteric, gram-negative bacillus with a polysaccharide capsule whose composition is used to classify various *Klebsiella* serotypes. Capsule serotypes K1 and K2 demonstrate increased resistance to intracellular destruction from immune cells and appear to predominate in cases of spread to other organs, particularly with ocular involvement [7], [8]. The bacteria’s ability to avoid phagocytosis among K1 and K2 serotypes has been demonstrated to be enhanced in patients with diabetes mellitus patients with poor glycemic control [7]. 

Cases of KPEE classically occur in patients with pyogenic liver abscess and/or diabetes mellitus. Although rare, it has also been reported in patients with a history of extremity cellulitis [9], urinary tract infection [4], [10], and intravenous drug use [11]. *Klebsiella* endophthalmitis can also develop from exogenous means such as penetrating trauma to the eye [9], a perforated corneal ulcer [12], and after intraocular surgery. 

Extension of bacterial endophthalmitis into the orbit is seldom reported, and typically occurs in patients with underlying immunosuppression [6]. There has been only one reported case of concurrent endophthalmitis and orbital cellulitis from *K. pneumoniae*, in which the patient was found to have a liver abscess [13]. To our knowledge, this is the first reported case of *K. pneumoniae* endogenous endophthalmitis with orbital cellulitis in a patient without a liver abscess.

Due to the absence of an extraocular nidus of infection (i.e. liver abscess), the source of *K. pneumoniae* remains unclear. It is worth mentioning that the patient had a recent history of lower extremity cellulitis with incision and drainage of multiple abscesses. Blood cultures at that time, however, grew *Streptococcal* sp. It is the opinion of the authors that the most likely source is transient bacteremia from intravenous drug abuse. 

Additionally, it is unclear if the infection initially inoculated the orbit and spread inwardly; or conversely, if the infection began within the globe and extended outward to the orbital structures. The authors believe that due to the disproportionate severity of orbital inflammation in relation to endophthalmitis upon initial presentation, it is possible that the infection primarily began in the orbit and spread inwardly, to the eye. However, this remains unclear.

## Conclusion

*Klebsiella pneumoniae* endogenous endophthalmitis with concurrent orbital cellulitis is a visually devastating and potentially deadly infection. Early and aggressive medical therapy and vitrectomy are often inadequate, and evisceration or enucleation is often necessary to control the infection. Eye care providers should always remember to perform a dilated fundus exam or posterior segment ultrasound in cases of orbital cellulitis to rule out concurrent endophthalmitis.

## Notes

### Competing interests

The authors declare that they have no competing interests.
